# Association between obstructive sleep apnea hypopnea syndrome and arteriosclerosis in patients with type 2 diabetes mellitus: mediating effect of blood pressure

**DOI:** 10.3389/fendo.2025.1510737

**Published:** 2025-02-12

**Authors:** Xinshui Wang, Xiaolin Huang, Yuexian Xing, Xiaohong Jiang, Fei Hua

**Affiliations:** Department of Endocrinology, The Third Affiliated Hospital of Soochow University, Changzhou, China

**Keywords:** obstructive sleep apnea hypopnea syndrome, type 2 diabetes mellitus, arteriosclerosis, mediation effect, cardiovascular risk, vascular health

## Abstract

**Objective:**

This study aims to explore the relationship between Obstructive Sleep Apnea Hypopnea Syndrome (OSAHS) and arteriosclerosis in type 2 diabetes mellitus (T2DM) patients and to evaluate the mediating effect of blood pressure in this process.

**Methods:**

A total of 411 T2DM patients admitted to the Third Affiliated Hospital of Soochow University from January 2021 to December 2023 were selected and divided into the arteriosclerosis group (n = 299) and the non-arteriosclerosis group (n = 112) based on brachial-ankle pulse wave velocity (ba-PWV). General clinical data, metabolic indicators, and sleep-related parameters were collected. The relationship between the apnea-hypopnea index (AHI) and arteriosclerosis was analyzed using univariable and multivariable logistic regression models, while a generalized additive model (GAM) was applied for curve fitting. A segmented regression model was used to explain nonlinearity, and subgroup analysis was conducted to assess interactions. Finally, a mediation effect model evaluated AHI’s direct and indirect effects on arteriosclerosis.

**Results:**

The AHI of the arteriosclerosis group was significantly higher than that of the non-arteriosclerosis group (P < 0.001). In the unadjusted, partially adjusted, and fully adjusted regression analyses, elevated AHI significantly increased the risk of arteriosclerosis (P < 0.05). Curve fitting indicated a near-linear positive correlation (P = 0.033). The segmented regression model showed that when AHI < 8.8 events/hour, the risk of arteriosclerosis significantly increased with higher AHI (P = 0.008), but the risk increase was not significant when AHI > 8.8 events/hour (P = 0.124). There was no significant interaction between AHI and blood pressure-related index subgroup indicators (P > 0.05). Mediation analysis revealed that systolic blood pressure (SBP), diastolic blood pressure (DBP), and mean arterial pressure (MAP) had significant mediating effects on the relationship between AHI and arteriosclerosis (P < 0.05), but the direct effect of AHI on arteriosclerosis was not significant (P > 0.05).

**Conclusion:**

OSAHS severity elevates arteriosclerosis risk in T2DM patients. Blood pressure is a partial intermediary in this effect.

## 1 Introduction

Type 2 diabetes mellitus (T2DM) is a common chronic metabolic disease (1, 2). Chronic complications are the main causes of adverse outcomes in T2DM patients, including blindness, end-stage renal disease, limb amputation, and cardiovascular diseases (3). Among these, cardiovascular diseases are the most severe, being a major cause of disability and death in patients (4). Obstructive sleep apnea hypopnea syndrome (OSAHS) is a clinical syndrome mainly characterized by repeated hypoventilation and respiratory interruption during sleep due to various reasons, resulting in hypoxemia, hypercapnia, and sleep structure disorders (5, 6). As an emerging risk factor for cardiovascular disease, studies have shown that the presence and severity of OSAHS are associated with a higher prevalence of cardiovascular diseases, especially atherosclerosis, hypertension, stroke, and heart failure (7), which severely impacts the quality of life and causing negative effects on both physical and mental health. In recent years, it has gradually attracted attention.

Studies have pointed out that sleep apnea and related hypoxemia can harm the cardiovascular system through mechanisms such as sympathetic nervous system activation, oxidative stress, endothelial dysfunction, systemic inflammation, and lipid peroxidation (8). A meta-analysis by Wang et al. (9) of 12 prospective cohort studies found that, compared to healthy individuals, patients with severe OSAHS had a relative risk of 1.79 for cardiovascular adverse events. Additionally, for every 10-unit increase in AHI, the risk of cardiovascular diseases increased by 17%. A follow-up study conducted by Souza et al. (10) demonstrated that in a cohort with an average age of 48 years, mild, moderate, or severe OSAHS were independently associated with atherosclerosis. However, some researchers believe that OSAHS may not be an independent factor directly causing atherosclerosis. Patients typically exhibit a range of cardiovascular risk factors, which can accelerate the progression of atherosclerosis, even causing vascular lesions in the early stages of the disease (11, 12). A study has shown that T2DM patients with OSAHS develop arteriosclerosis earlier (13). Adderley et al. (14) also proposed that OSAHS in T2DM patients significantly increases the risk of cardiovascular diseases. A longitudinal study spanning 4.9 years on T2DM patients revealed that sleep-disordered breathing predicts adverse cardiovascular events, with a hazard ratio of 1.9 (15). It appears that the coexistence of OSAHS and T2DM seems to have a synergistic effect, elevating the risk of cardiovascular events in these patients. However, some studies have also shown that the association between OSAHS and cardiovascular disease is not strong in T2DM patients (16). A study indicates that, in T2DM patients, although the severity of OSAHS was not significantly associated with adverse cardiovascular events, the cardiovascular disease risk still trends upward in mild OSAHS (17). This study will focus on mild patients, aiming to confirm this increased risk trend so that doctors and patients can identify it earlier and take intervention measures.

Research shows that OSAHS, especially moderate-to-severe OSAHS, is closely related to hypertension (18). Hypoxemia in OSAHS patients triggers excessive activation of the sympathetic nervous system, leading to elevated blood pressure, which induces and accelerates atherosclerosis (19). Therefore, hypertensive patients with OSAHS have a higher risk of adverse cardiovascular events (20). At the same time, hypertension plays a crucial role in T2DM-related complications. Beyond blood glucose control, systolic blood pressure (SBP) has been proven to have independent and additive effects on microvascular and macrovascular complications in T2DM patients (21). Hypertension is likely to mediate, at least to some extent, the relationship between OSAHS and adverse cardiovascular events in T2DM patients, but validation with large sample data is still needed.

Given this, this study proposes the following hypotheses: First, OSAHS may affect arteriosclerosis in T2DM patients, and the arteriosclerosis risk gradually increases with the severity of OSAHS. Secondly, mild OSAHS can also contribute to the arteriosclerosis risk in T2DM patients. Thirdly, the severity of OSAHS may have direct and indirect impacts on arteriosclerosis in T2DM patients, with blood pressure as an intermediary variable in this relationship.

## 2 Materials and methods

### 2.1 Clinical data

**Figure 1:**
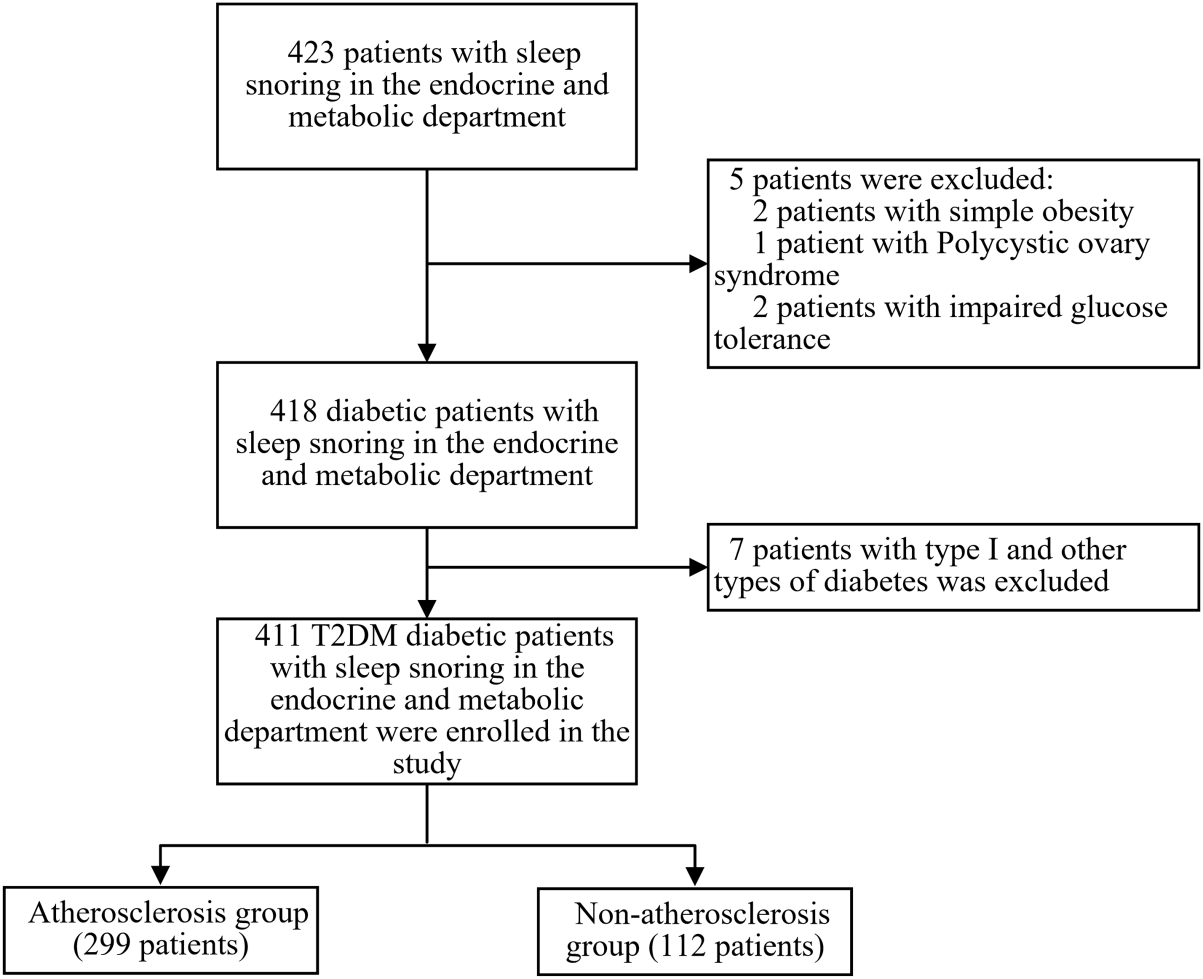
Flow chart of patient enrollment.

This is a single-center, retrospective, cross-sectional study. 423 patients with clinical sleep-related snoring who were hospitalized in the Department of Endocrinology and Metabolism at the Third Affiliated Hospital of Soochow University from January 2021 to December 2023 were recruited. Inclusion criteria: 1) Patients diagnosed with T2DM based on the 1999 WHO diabetes diagnostic criteria (22); 2) Patients whose snoring during sleep was observed by themselves or their family members. Exclusion criteria: 1) Patients under 18 years old; 2) Patients with impaired glucose tolerance, type 1, or other types of diabetes; 3) Patients with blood system diseases, heart failure, acute myocardial infarction, or severe arrhythmia, chronic obstructive pulmonary disease (COPD) or other lung diseases causing significant hypoxemia, cirrhosis or other diseases causing abnormal liver function, chronic kidney disease (CKD) Stage 4-5 and malignancies. Ultimately, 411 T2DM patients with OSAHS were included in the study. This study followed the principles of the Declaration of Helsinki and was approved by the Ethics Committee of our hospital [ethics number (2024): KD 006]. Since all patients were anonymized, informed consent was not required. See **Figure 1** for the enrollment flowchart.

### 2.2 Diagnosis

#### 2.2.1 Type II diabetes

The diagnosis of T2DM is based on the diagnostic criteria proposed by the WHO Expert Committee on Diabetes (1999) (22) and can be confirmed if any of the following conditions are met: random blood glucose ≥ 11.1 mmol/L in symptomatic individuals or fasting blood glucose (FPG, fasting for more than 8 hours) ≥ 7.0 mmol/L; or 2-hour blood glucose during oral glucose tolerance test (OGTT) ≥ 11.1 mmol/L. The following day, asymptomatic individuals must retest the following day to confirm the diagnosis. The diagnosis should consider the patient’s symptoms, islet function, and other comprehensive factors.

#### 2.2.2 OSAHS and simple snoring

The diagnosis of OSAHS and simple snoring is based on the diagnostic criteria outlined in the “Guidelines for the Diagnosis and Treatment of Obstructive Sleep Apnea Hypopnea Syndrome” (2011 Revised Edition) (23), primarily relying on the patient’s medical history, clinical signs, and polysomnography (PSG) monitoring data. Clinically, patients exhibit typical symptoms such as snoring and irregular breathing during nighttime sleep and daytime symptoms like excessive sleepiness. If PSG monitoring shows an AHI (Apnea-Hypopnea Index) of ≥ 5 times per hour during 7 hours of sleep each night, OSAHS is diagnosed. Additionally, if the patient has no daytime symptoms but has an AHI ≥ 5 times per hour and at least one major organ damage, OSAHS is also diagnosed. Simple snoring is diagnosed if the patient snores varying degrees at night, has an AHI < 5 times per hour, and has no daytime symptoms (24). OSAHS is further classified into mild (5 ≤ AHI < 15 times/hour), moderate (15 ≤ AHI < 30 times/hour), and severe (AHI ≥ 30 times/hour), according to AHI. To improve the statistical power and clinical significance of the analysis, this study combined mild and moderate OSAHS into one group (5 ≤ AHI < 30 times/hour) to better investigate the impact of OSAHS severity on arteriosclerosis.

#### 2.2.3 Arteriosclerosis

To avoid geographical and racial biases to a certain extent, this study primarily refers to guidelines and research currently targeted at Asian populations (25, 26). According to the experts’ consensus (27), this study defines arteriosclerosis as a brachial-ankle pulse wave velocity (ba-PWV) ≥ 1400 cm/s.

The formula for calculating ba-PWV is: Ba-PWV (cm/s) = s/Δt.

Where *s* is the distance between the brachial and ankle arteries (cm), and *Δt* is the pulse wave transmission time (s).

#### 2.2.4 Hypertension

According to the “Guidelines for the Prevention and Treatment of Hypertension in China” (28), the diagnostic criteria are as follows: Blood pressure measured on three separate occasions on different days with systolic pressure ≥ 140 mmHg and/or diastolic pressure ≥ 90 mmHg without the use of antihypertensive medications; or if the patient has a history of hypertension and is currently taking antihypertensive medications with well-controlled blood pressure.

### 2.3 Laboratory test

#### 2.3.1 General information

General information about the enrolled T2DM patients was collected, including age, gender, height, weight, hip circumference, waist circumference, blood pressure, smoking history, history of hypertension, history of diabetes, history of myocardial infarction, and history of stroke. The duration of diabetes was recorded from the first diagnosis of T2DM to the day of the visit (in months), and the duration of hypertension from the first diagnosis to the day of the visit (in years). The following indices were calculated based on formulas: BMI = weight (kg)/height (m²); WHR = waist circumference (WC, cm)/hip circumference (HC, cm); WHtR = waist circumference (cm)/height (cm).

#### 2.3.2 Glucose and lipid metabolism indicators

After patients were admitted, venous blood was collected the next morning after an overnight fast of 8-10 hours to measure glucose and lipid metabolism indicators. An automatic biochemical analyzer (Beckman Coulter AU5800, Brea, CA, USA) was used to measure FPG and lipid levels. FPG was measured using the glucose oxidase method, while total cholesterol (TC), triglyceride (TG), high-density lipoprotein-cholesterol (HDL-C), and low-density lipoprotein-cholesterol (LDL-C) were measured using the photometric colorimetric method. Apolipoprotein-A1(Apo-A1) and Apolipoprotein-B(Apo-B) were measured using the immunoturbidimetric method. HbA1c was measured using high-performance liquid chromatography (D-10 system, Bio-Rad, USA), and fasting C-peptide was measured using electrochemiluminescence immunoassay (Roche Cobas8000, Indianapolis, IN, USA). Additionally, the following indices were calculated: ① LDL-C/Apo-B, ② Apo-B/Apo-A1, ③ TyG=ln[TG(mg/dL)×FBG(mg/dL)/2] (29), ④ AIP = log[TG (mmol/L)/HDL-C (mmol/L)].

#### 2.3.3 Sleep indicators

Polysomnography monitoring: Participants were instructed to refrain from consuming alcohol, theophylline, caffeine, and sedative or hypnotic medications for 48 hours before the monitoring. Sleep parameters were obtained by using the Somnostar 4000 (Sensor Medics Inc, USA) polysomnography monitor for continuous monitoring for 7 hours or more. The monitored data included AHI, oxygen desaturation index (ODI), lowest blood oxygen saturation (L-pO2), average blood oxygen saturation (M-pO2), and average heart rate (M-HR).

#### 2.3.4 Ba-PWV measurement

Patients were asked to rest for more than 5 minutes before the measurement and then lie supine on the examination bed, with both hands placed at their sides, maintaining a relaxed state (27). A well-trained and experienced doctor measured and recorded the mean pulse pressure (PP), MAP, and ba-PWV data using an arterial stiffness detector (OMRON HBP-8000).

#### 2.3.5 Blood pressure measurement

Blood pressure measurements were obtained before ba-PWV measurements. The patients emptied their bladders and rested quietly for 30 minutes, avoiding emotional fluctuations, strenuous exercise, and the intake of ingredients that can cause blood pressure fluctuations (including theophylline, caffeine, nicotine, etc.). The patient sits with the right arm on the table, maintaining the elbow bent and at heart level. An electronic sphygmomanometer is used to measure and record SBP and DBP.

## 3 Statistical methods

Statistical analysis was performed using R software (version 4.2.0; http://www.R-project.org). The main toolkits include `stats`, `mgcv`, `lmtest`, and `mediation`. Descriptive statistics were used for general clinical data, glucose and lipid metabolism indicators, and sleep-related parameters. Continuous variables were described using means (standard deviation) or medians (interquartile range), and comparisons between groups were made using the t-test or Mann-Whitney U test. Categorical variables were described using frequencies (percentages), and comparisons between groups were made using the χ² test or Fisher’s exact test.

A generalized linear model with a logit link was used to test AHI’s independent and combined effects on the arteriosclerosis status. The predictor of univariate logistic regression analysis was the independent variable AHI, which was used to preliminarily explore its relationship with arteriosclerosis. In the multivariate logistic regression analysis, we used a stepwise adjustment approach: the initial adjustment model incorporated key confounders recognized in the literature (e.g., sex, age, BMI, smoking history, and duration of diabetes); the fully adjusted model was based on the preliminary adjusted model and further included potential confounders that may significantly affect the results (i.e., variables that changed the estimated values ​​of AHI and atherosclerosis risk by more than 10% or were significantly associated with atherosclerosis risk, P <0.1). We calculated unadjusted and adjusted estimates using both exact and asymptotic methods and finally calculated Regression coefficients (β), odds ratios (OR), and their 95% confidence intervals (CI).

A generalized additive model (GAM) was used to fit a smoothed curve for the relationship between AHI and arteriosclerosis, further exploring the nonlinear relationship. Confounders were fully adjusted in the model, and the trend between AHI and arteriosclerosis was observed through curve-fitting results. To evaluate the threshold effect of AHI on arteriosclerosis, this study adopted a segmented logistic regression model and used the log-likelihood ratio test to compare the goodness of fit between two nested models to determine whether an inflection point needed to be introduced. When the P value was significant (P < 0.05), it indicated a significant threshold effect in the model, thus supporting the selection of the segmented model.

A stratified analysis method assessed the interaction between AHI and arteriosclerosis across blood pressure subgroups (hypertension history, SBP, DBP, PP, MAP). The regression coefficients and P values between different subgroups were compared to determine whether significant interaction effects existed.

A mediation effect model was employed to evaluate whether the effect of AHI on arteriosclerosis was indirectly mediated through mediating variables such as blood pressure (SBP, DBP, MAP). The model adjusted for confounding variables such as gender, age, BMI, smoking history, and diabetes duration, then calculated the direct and indirect effects and their proportions. The significance of the mediation effect was tested by the Bootstrap method using the mediation package (version 4.0) of R software, with a sampling frequency of 1000 times, and the percentile method was used to generate the 95% confidence interval of the effect value. All statistical analyses were conducted using a two-sided test, with P < 0.05 considered statistically significant.

## 4 Results

Among the 411 T2DM patients included in the study, ages ranged from 18 to 88 years (mean age 52.6 ± 14.9 years), with 276 males (67.2%) and 135 females (32.8%). Based on the criteria for arteriosclerosis, patients were divided into the arteriosclerosis group (299 patients) and the non-arteriosclerosis group (112 patients). Based on AHI, there were 44 patients with simple snoring, 254 with mild-moderate OSAHS, and 113 with severe OSAHS.

### 4.1 Comparison of general clinical data and laboratory indicators between the arteriosclerosis group and non-arteriosclerosis group

There were no significant differences between the two groups in terms of gender composition, smoking history, BMI, WC, HC, WHR, WHtR, and the prevalence of myocardial infarction (P > 0.05). However, patients in the arteriosclerosis group had significantly higher age, diabetes duration, hypertension duration, hypertension prevalence, stroke prevalence, SBP, DBP, PP, and MAP compared to the non-arteriosclerosis group (P < 0.05). For glucose and lipid metabolism indicators, the arteriosclerosis group had higher Apo-A1 levels (P < 0.05), while Apo-B, LDL-C, HbA1c, and Apo-B/Apo-A1 levels were lower than those in the non-arteriosclerosis group (P < 0.05). There were no significant differences between the two groups in FPG, C-peptide, TC, TG, HDL-C, LDL-C/Apo-B, TyG, and AIP levels (P > 0.05). Additionally, there were no significant differences in the sleep monitoring indicators, L-pO2, M-pO2, and M-HR (P > 0.05). However, the AHI and ODI were significantly higher in the arteriosclerosis group compared to the non-arteriosclerosis group (P < 0.05) (see **Table 1**).

**Table 1:** Comparison of clinical characteristics between non-atherosclerosis group and atherosclerosis group.

	Total (n=411)	Non-atherosclerosis group (n=112)	Atherosclerosis group (n=299)	P-value
Age (years)	52.6 (14.9)	41.9 (12.6)	56.7 (13.6)	<0.001
Gender				0.109
Female	135 (32.8%)	30 (26.8%)	105 (35.1%)	
Male	276 (67.2%)	82 (73.2%)	194 (64.9%)	
Smoking history	93 (22.6%)	22 (19.6%)	71 (23.7%)	0.376
BMI (kg/m^2^)	26.4 (4.6)	27.0 (5.3)	26.2 (4.3)	0.100
Diabetes duration (months)	60 (2–120)	12 (1–60)	84 (6–144)	<0.001
WC (cm)	95 (12)	97 (14)	95 (11)	0.120
HC (cm)	100 (9)	101 (10)	100 (9)	0.171
WHR	0.95 (0.06)	0.96 (0.07)	0.95 (0.06)	0.436
WHtR	0.57 (0.07)	0.57 (0.07)	0.57 (0.06)	0.643
Hypertension history	234 (56.9%)	35 (31.2%)	199 (66.6%)	<0.001
Hypertension duration (years)	1 (0–10)	0 (0–1)	5 (0–10)	<0.001
Myocardial infarction	6 (1.5%)	0 (0.0%)	6 (2.0%)	0.196
Stroke	26 (6.3%)	1 (0.9%)	25 (8.4%)	0.006
SBP (mmHg)	134 (18)	124 (13)	138 (18)	<0.001
DBP (mmHg)	82 (10)	78 (9)	83 (11)	<0.001
PP (mmHg)	52 (12)	46 (7)	54 (12)	<0.001
MAP (mmHg)	99 (12)	93 (10)	101 (12)	<0.001
Laboratory indicators				
FPG (mmol/L)	7.9 (6.5-9.6)	8.4 (2.7)	8.6 (3.2)	0.519
C peptide (pmol/L)	605.9 (410.9-875.5)	585.15 (410.62-883.05)	608.40 (411.45-866.20)	0.832
Apo-A1 (g/L)	1.16 (0.27)	1.11 (0.27)	1.18 (0.27)	0.020
Apo-B (g/L)	0.99 (0.33)	1.05 (0.29)	0.96 (0.34)	0.017
TC (mmol/L)	4.73 (1.37)	4.91 (1.20)	4.66 (1.43)	0.107
TG (mmol/L)	1.79 (1.25-2.60)	1.77 (1.25-2.55)	1.81 (1.26-2.64)	0.820
HDL-C (mmol/L)	1.06 (0.28)	1.01 (0.31)	1.07 (0.27)	0.058
LDL-C (mmol/L)	2.81 (1.01)	2.98 (0.90)	2.75 (1.04)	0.036
HbA1c (%)	10.0 (2.4)	10.4 (2.6)	9.8 (2.4)	0.036
LDL-C/Apo-B	2.89 (0.82)	2.83 (0.32)	2.92 (0.95)	0.358
Apo-B/Apo-A1	0.84 (0.63-1.10)	0.98 (0.73-1.21)	0.78 (0.62-1.04)	<0.001
TyG	9.42 (0.76)	9.41 (0.77)	9.43 (0.76)	0.851
AIP	0.24 (0.04-0.43)	0.26 (0.07-0.44)	0.23 (0.04-0.43)	0.500
AHI (events/h)	17.8 (9.2-31.2)	13.3 (5.8-26.9)	19.0 (10.6-34.1)	<0.001
ODI (events/h)	20.0 (10.6-32.3)	15.2 (7.4-29.6)	21.2 (12.1-32.8)	0.006
L-pO2 (%)	80 (10)	81 (12)	80 (10)	0.280
M-pO2 (%)	94 (2)	94 (3)	94 (2)	0.441
M-HR (bpm)	69.8 (9.3)	69.4 (9.3)	70.0 (9.4)	0.553
Ba-PWV (cm/s)	1637 (368)	1271 (102)	1774 (337)	<0.001

Data in the table: Mean(SD) Median (Q1-Q3)/N(%).

BMI, Body mass index; WC, Waist circumference; HC, Hips circumference; WHR, Waist to hip ratio; WHtR, Waist to height ratio; FPG, fasting blood glucose; Apo-A1, Apolipoprotein-A1; Apo-B, Apolipoprotein-B; TC, Total cholesterol; TG, Triglyceride; HDL-C, High-density lipoprotein-cholesterol; LDL-C, Low-density lipoprotein-cholesterol; HbA1c, Glycated hemoglobin; SBP, Systolic blood pressure; DBP, Diastolic blood pressure; PP, Pulse pressure; MAP, Mean arterial blood pressure; TyG, Triglyceride-Glucose Index; AIP, Atherogenic index of plasma; AHI, Apnea-hypopnea index; ODI, Oxygen desaturation index; L-pO2, Lowest blood oxygen saturation; M-pO2, Mean blood oxygen saturation; M-HR, Mean heart rate; Ba-PWV, Brachial-ankle pulse wave velocity.

### 4.2 Logistic regression analysis of the impact of AHI on arteriosclerosis

**Table 2** presents the results of univariable and multivariable logistic regression analyses for continuous AHI variables and the three AHI subgroups. The unadjusted model corresponds to univariable logistic regression analysis, while the initially adjusted model includes adjustments for gender, age, BMI, smoking history, and diabetes duration. The fully adjusted model additionally includes hypertension history, hypertension duration, DBP, PP, and ODI.

**Table 2:** Multiple regression equation analysis of AHI on arteriosclerosis.

Exposure	Unadjusted Model		Initially adjusted model		Fully adjusted model	
	OR (95%CI)	P- value	OR (95%CI)	P- value	OR (95%CI)	P- value
AHI (events/h)	1.021 (1.006, 1.036)	0.005	1.018 (1.000, 1.035)	0.045	1.052 (1.004, 1.102)	0.033
Gender*	0.676 (0.418, 1.093)	0.110	1.003 (0.544, 1.850)	0.992	1.085 (0.555, 2.121)	0.811
Age (years)	1.083 (1.063, 1.104)	<0.001	1.081 (1.054, 1.108)	<0.001	1.068 (1.037, 1.099)	<0.001
BMI (kg/m^2^)	0.962 (0.919, 1.008)	0.102	1.042 (0.976, 1.112)	0.220	0.991 (0.918, 1.069)	0.812
Smoking history	1.274 (0.745, 2.179)	0.377	1.134 (0.597, 2.156)	0.701	1.359 (0.675, 2.735)	0.390
Diabetes duration (months)	1.010 (1.006, 1.014)	<0.001	1.004 (0.999, 1.008)	0.103	1.004 (1.000, 1.009)	0.071
Hypertension history	4.378 (2.747, 6.978)	<0.001	——	——	1.120 (0.518, 2.424)	0.773
Hypertension duration (years)	1.149 (1.094, 1.207)	<0.001	——	——	1.028 (0.957, 1.105)	0.447
DBP (mmHg)	1.059 (1.033, 1.085)	<0.001	——	——	1.077 (1.041, 1.115)	<0.001
PP (mmHg)	1.086 (1.058, 1.114)	<0.001	——	——	1.054 (1.019, 1.090)	0.002
ODI(events/h)	1.009 (0.997, 1.021)	0.136	——	——	0.965 (0.927, 1.003)	0.073
AHI groups						
<5	1.0		1.0		1.0	
5≤AHI<30	3.826 (1.978, 7.401)	<0.001	3.908 (1.754, 8.707)	<0.001	3.334 (1.379, 8.059)	0.008
≥30	5.443 (2.554, 11.600)	<0.001	5.634 (2.081, 15.255)	<0.001	4.855 (1.218, 19.353)	0.025
P for trend	<0.001		0.002		0.017	
Gender*	0.676 (0.418, 1.093)	0.110	0.902 (0.483, 1.685)	0.747	1.053 (0.534, 2.076)	0.881
Age (years)	1.083 (1.063, 1.104)	<0.001	1.076 (1.050, 1.104)	<0.001	1.065 (1.034, 1.097)	<0.001
BMI (kg/m^2^)	0.962 (0.919, 1.008)	0.102	1.026 (0.961, 1.095)	0.446	0.978 (0.908, 1.054)	0.560
Smoking history	1.274 (0.745, 2.179)	0.377	1.190 (0.624, 2.270)	0.597	1.354 (0.676, 2.710)	0.392
Diabetes duration (months)	1.010 (1.006, 1.014)	<0.001	1.004 (1.000, 1.009)	0.073	1.005 (1.000, 1.010)	0.042
Hypertension history	4.378 (2.747, 6.978)	<0.001	——	——	1.269 (0.591, 2.728)	0.541
Hypertension duration (years)	1.149 (1.094, 1.207)	<0.001	——	——	1.018 (0.948, 1.093)	0.627
DBP (mmHg)	1.059 (1.033, 1.085)	<0.001	——	——	1.073 (1.037, 1.111)	<0.001
PP (mmHg)	1.086 (1.058, 1.114)	<0.001	——	——	1.051 (1.016, 1.087)	0.004
ODI (events/h)	1.009 (0.997, 1.021)	0.136	——	——	0.987 (0.967, 1.007)	0.201

Unadjusted model: no adjustments.

Initially adjusted model: adjusted for gender, age, BMI, smoking history, and diabetes duration.

Fully adjusted model: adjusted for gender, age, BMI, smoking history, diabetes duration, hypertension history, hypertension duration, DBP, PP, and ODI.

AHI, Apnea-hypopnea index.

*Females are used as the reference group.

In the unadjusted, initially adjusted, and fully adjusted regression models for continuous AHI variables, an increase in AHI was associated with an increased risk of arteriosclerosis, with OR values of 1.021, 1.018, and 1.052, respectively, all of which were statistically significant (all P < 0.05). For the three AHI subgroups, increased AHI levels were also significantly associated with an increased risk of arteriosclerosis (all P for trend < 0.05).

### 4.3 Smoothing curve fitting

**Figure 2:**
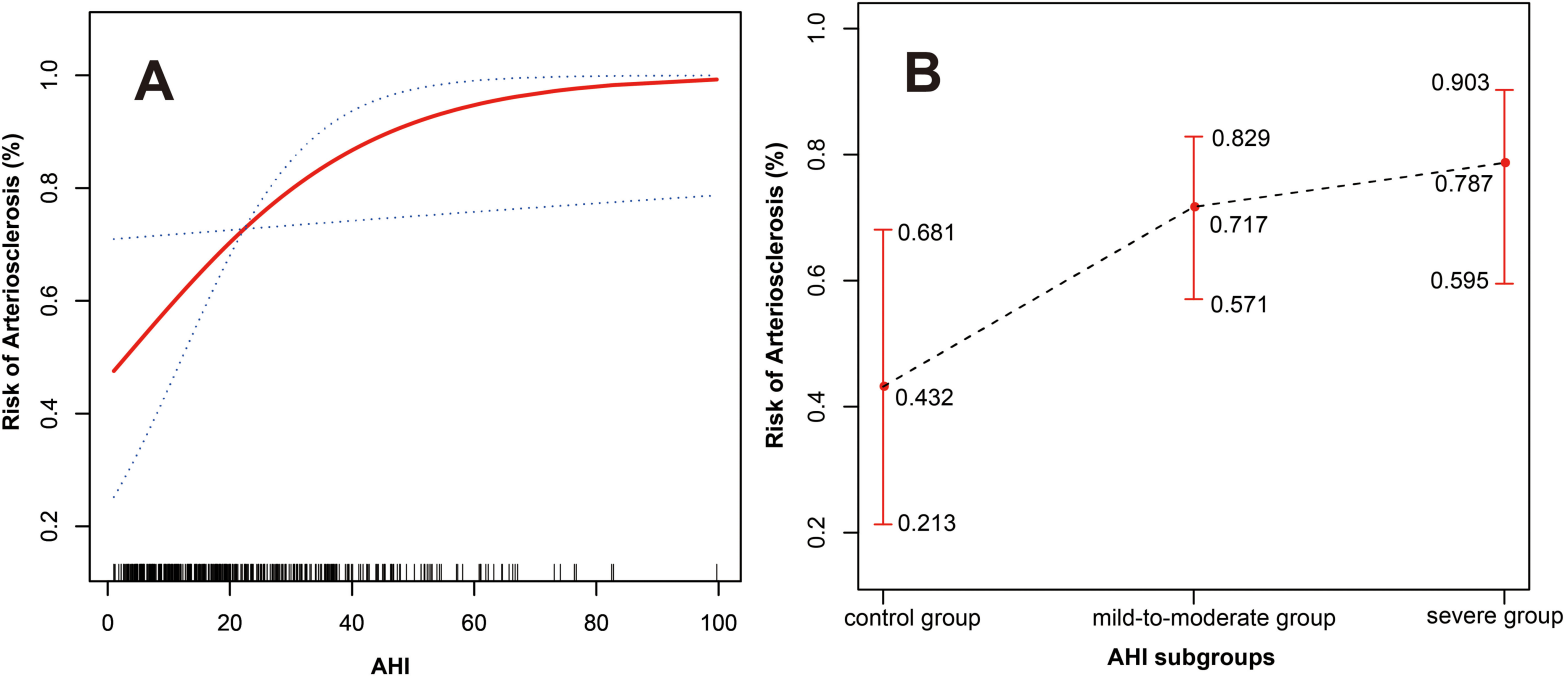
**(A)** The relationship between AHI and arteriosclerosis (the red solid line represents the fitted curve between AHI and the risk of arteriosclerosis; the blue dashed lines represent the 95% confidence interval). **(B)** The relationship between the three AHI subgroups and arteriosclerosis (the black dashed line represents the fitted curve between the three AHI subgroups and the risk of arteriosclerosis; the red lines represent the 95% confidence interval). Adjusted variables: gender, age, BMI, smoking history, diabetes duration, hypertension history, hypertension duration, DBP, PP, and ODI.

Curve fitting showed that after adjusting for gender, age, BMI, smoking history, diabetes duration, hypertension history, hypertension duration, DBP, PP, and ODI, the risk of arteriosclerosis gradually increased with higher AHI levels, displaying a near-linear positive correlation (degrees of freedom = 1.000, χ² = 4.571, P = 0.033) (see **Figure 2A**).

For the three AHI subgroups, after fully adjusting for confounding variables, there was also a near-linear relationship between different AHI levels and the risk of arteriosclerosis. As AHI levels increased, the risk of arteriosclerosis increased to 0.432 (95% CI: 0.213-0.681), 0.717 (95% CI: 0.571-0.829), and 0.787 (95% CI: 0.595-0.903) respectively (see **Figure 2B**).

### 4.4 Threshold effect

The segmented logistic regression model was used to assess whether there is a threshold effect in the fitted curve. The results showed that when the AHI was at the inflection point of 8.8 events/h, the log-likelihood ratio test suggested that the segmented logistic regression model was significantly better than the model without the inflection point (P=0.029), indicating the existence of a significant threshold effect. When AHI < 8.8 events/h, the arteriosclerosis risk significantly increased with rising AHI (OR = 1.255, 95% CI: 1.061-1.485, P = 0.008). However, when AHI > 8.8 events/h, the change in the arteriosclerosis risk with increasing AHI was not significant (P = 0.124) (see **Table 3**). This result suggests that even a mild increase in AHI may significantly impact atherosclerosis risk.

**Table 3:** Nonlinear relationship between AHI and arteriosclerosis.

Outcome:	OR (95%CI)	P-value
Model I		
Linear effect	1.052 (1.004, 1.102)	0.033
Model II		
Inflection point (K)	8.8 events/h	
< K segment effect 1	1.255 (1.061, 1.485)	0.008
> K segment effect 2	1.038 (0.990, 1.089)	0.124
Effect difference between 2 and 1	0.827 (0.696, 0.983)	0.031
Equation prediction value at the inflection point	1.202 (0.831, 1.573)	
Log-likelihood ratio test	0.029	

Adjusted variables: gender, age, BMI, smoking history, diabetes duration, hypertension history, duration of hypertension, DBP, PP, and ODI.

### 4.5 Stratified analysis

**Figure 3:**
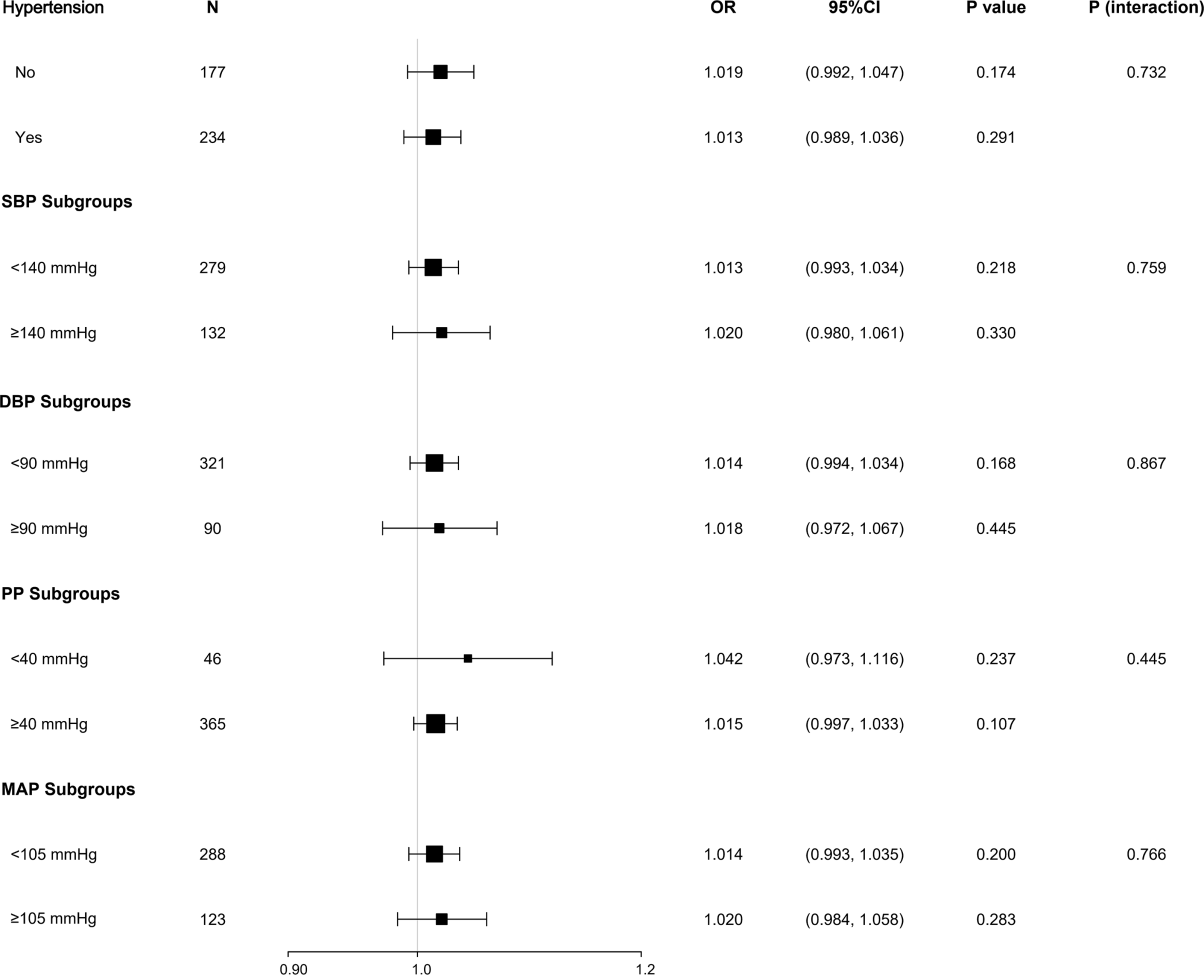
Stratified analysis of the association between AHI and arteriosclerosis. Subgroup analysis based on hypertension history and blood pressure indices (SBP, DBP, PP, MAP) showed no significant interaction effects (P for interaction > 0.05). Adjusted variables: gender, age, BMI, smoking history, diabetes duration.

After adjusting for gender, age, BMI, smoking history, and duration of diabetes, the relationship between AHI and arteriosclerosis was compared across different subgroups based on hypertension history, SBP, DBP, PP, and MAP. The results showed that neither the history of hypertension nor the different blood pressure indicator subgroups significantly altered the association between AHI and arteriosclerosis (all P > 0.05), indicating no interaction effect (see **Figure 3**).

### 4.6 Mediation analysis of different blood pressure indicators on the relationship between AHI and arteriosclerosis

**Figure 4:**
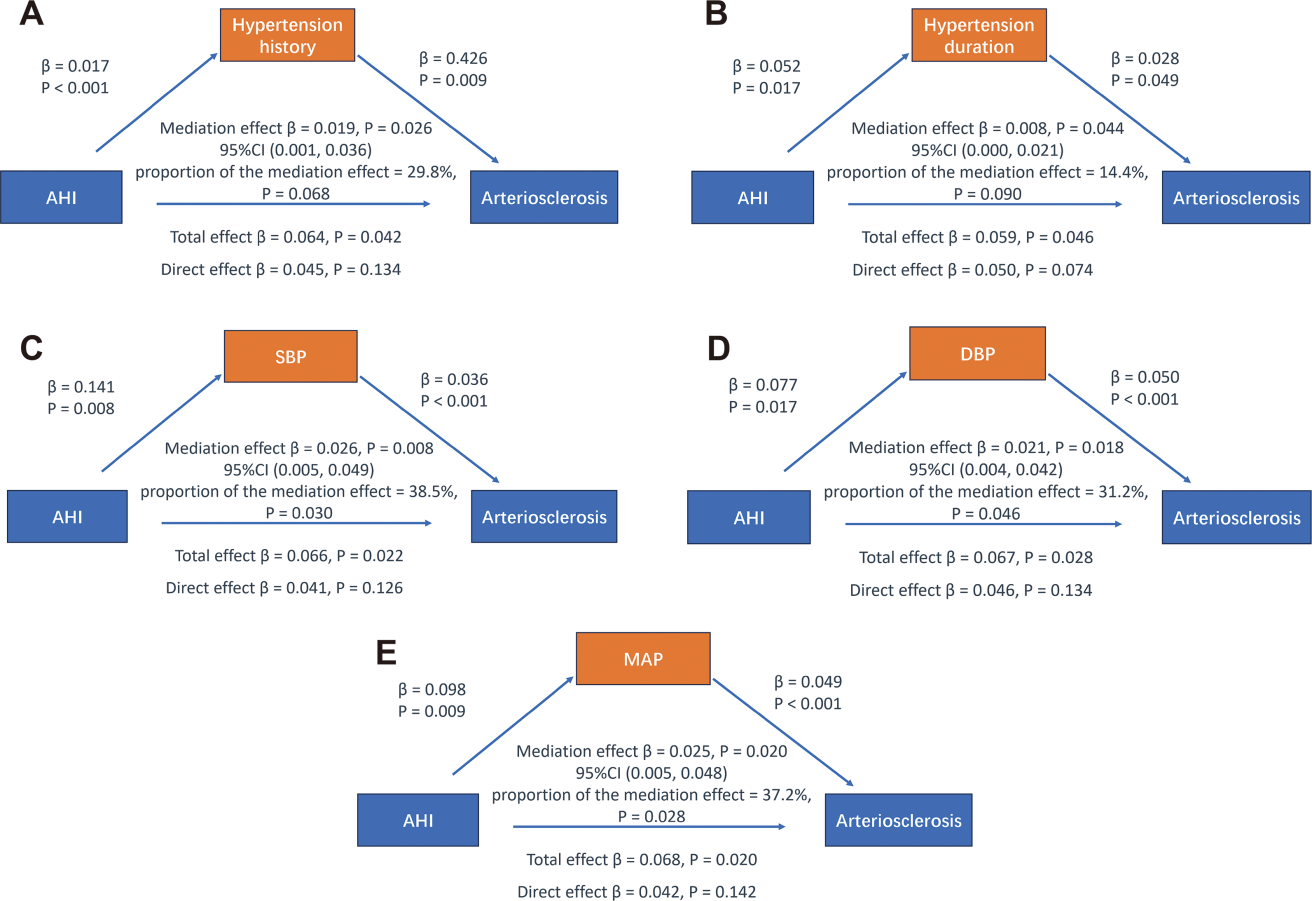
Mediation analysis of different blood pressure indicators on the relationship between AHI and arteriosclerosis. Adjusted variables: gender, age, BMI, smoking history, diabetes duration.

After adjusting for confounding factors such as gender, age, BMI, smoking history, and duration of diabetes, both hypertension history and duration of hypertension were found to have a mediating effect on the relationship between AHI and arteriosclerosis (β = 0.019, P = 0.026; β = 0.008, P = 0.044), though the proportion of the mediation effect was not significant (both P > 0.05). Additionally, the direct effect of AHI on arteriosclerosis was not significant (all P > 0.05) (see **Figures 4A, B**). These findings suggest that AHI may influence arteriosclerosis by affecting the presence and duration of hypertension, although the proportion of the mediating effect of hypertension history and duration was insignificant.

The results also showed that SBP, DBP, and MAP serve as mediating variables in the relationship between AHI and arteriosclerosis, with mediation proportions of 38.5% (β = 0.026, P = 0.008), 31.2% (β = 0.021, P = 0.018), and 37.2% (β = 0.025, P = 0.020), respectively. However, the direct effect of AHI on arteriosclerosis remained insignificant (all P > 0.05) (see **Figures 4C-E**). These findings suggest that changes in blood pressure levels largely mediate the relationship between AHI and arteriosclerosis. This may indicate that AHI indirectly contributes to increased arterial stiffness by influencing blood pressure indicators rather than having a direct effect.

## 5 Discussion

This study found a significant association between OSAHS and arteriosclerosis in individuals with T2DM. As the severity of OSAHS increases, the risk of arteriosclerosis also gradually rises. When AHI < 8.8 events/h, the increase in AHI significantly raises the risk of arteriosclerosis, indicating that even a slight elevation of AHI beyond the normal range (5 events/h) may begin to impact arteriosclerosis negatively. The study also revealed that blood pressure indicators mediate the process of OSAHS-related arteriosclerosis. This suggests that the effect of OSAHS on arteriosclerosis is partly exerted through blood pressure, highlighting the importance of actively controlling related risk factors in T2DM patients with OSAHS in clinical practice.

As a common sleep-breathing disorder, OSAHS has gradually attracted widespread attention. Increasing evidence suggests that OSAHS plays a significant role in the occurrence and progression of arteriosclerosis (30–33). OSAHS may disrupt the body’s metabolism and normal physiological rhythms through various pathways such as intermittent hypoxia, sympathetic activation, and the influence on the Hypothalamic-Pituitary-Adrenal (HPA) Axis, thereby advancing the course of arteriosclerosis (34). OSAHS and T2DM often coexist, and the prevalence of OSAHS is significantly higher in patients with T2DM compared to the general population (35). In T2DM patients with OSAHS, macrovascular and microvascular complications increase significantly (36). In a study involving 305 T2DM patients, it was found that AHI was associated with a 2.57-fold increase in stroke risk (37). Research by Adderley and Subramanian (14) showed that T2DM patients who developed OSAHS during follow-up had a significantly higher risk of peripheral neuropathy and atrial fibrillation. A study from the Da Qing Diabetes Research Center also indicated that T2DM patients with OSAHS are more prone to arteriosclerosis, and early intervention is recommended (13). In our study, the AHI and ODI levels were significantly higher in the arteriosclerosis group, indicating more severe sleep apnea and hypoxemia in this group. The results also showed that T2DM patients with atherosclerosis had higher Apo-A1 but lower Apo-B, LDL-C, Apo-B/Apo-A1, and HbA1c. We noted that despite being diagnosed with arteriosclerosis, patients might be at different stages and have received various degrees of treatments, such as a balanced diet, regular exercise, smoking cessation, alcohol restriction, effective glucose, and lipid-lowering treatments, causing observed lipid and glucose metabolism indicators to differ from expectations (38). In the future, we need larger samples, stricter grouping criteria, and more comprehensive variable controls to verify our findings. Further research showed that even mild OSAHS, compared to simple snoring, was associated with arteriosclerosis, and as OSAHS severity increased, so did the risk of arteriosclerosis. Notably, this correlation remains robust in the stratified analysis of different blood pressure indicators, and OSAHS remains an independent risk factor for arteriosclerosis. A study from Beijing indicated that ba-PWV was significantly elevated in T2DM patients with OSAHS, and after adjusting for age, gender, BMI, diabetes duration, blood glucose, blood pressure, blood lipids, and other factors, AHI remained an independent risk factor for increased ba-PWV (33). Drager and Queiroz (39) also found that when diabetes and OSAHS coexist, patients face a higher risk of arteriosclerosis, and the severity of OSAHS correlates with the severity of arteriosclerosis. However, some studies have pointed out that although there is a significant relationship between OSAHS and arteriosclerosis in T2DM patients, the severity of OSAHS is not necessarily related to the severity of arteriosclerosis (40–42). In our study, we did not conduct exhaustive and quantitative monitoring of patients’ dietary habits (such as meal structure, calorie intake, etc.) and medication use (including types, dosages, adherence, etc.), which increases the possibility of generating other unknown confounding effects and may have contributed to differences in the results.

This study also found a threshold effect between AHI and the risk of arteriosclerosis in T2DM patients. Compared to the conventional AHI classification (mild, moderate, severe), the threshold of 8.8 events/h is much lower than the commonly defined upper limit of mild OSAHS (15 events/h). This may suggest that in T2DM patients, even a slight increase in AHI beyond the normal range (5 events/h) could negatively impact arteriosclerosis. This finding highlights the importance of early identification and intervention in patients with mild OSAHS, as it may help reduce the risk of arteriosclerosis and associated cardiovascular events. This is especially important for T2DM patients, as they are already at an increased risk of cardiovascular disease due to diabetes itself. A domestic multicenter prospective study (43) found that T2DM and OSAHS synergistically affect arteriosclerosis. Although the severity of OSAHS was not significantly associated with the risk of major adverse cardiovascular events (MACE), there was an increasing trend of MACE risk in T2DM patients with mild OSAHS, similar to our findings. Severe OSAHS may involve more effective self-protective mechanisms, such as increased breathing or respiratory rate, to compensate for hypoxia, thereby reducing the risk of adverse events (44). A study of 131 T2DM patients found moderate-to-severe OSAHS triples the risk of cardiovascular disease compared to mild or absent OSAHS (17). Some researchers believe that mild OSAHS does not show significant changes in endothelial function and inflammatory indicators and may be considered a confounding variable that needs to be controlled in research (45). Therefore, we look forward to future studies that can further explore this threshold effect and provide a solid evidence base for its application in clinical practice.

In T2DM patients, the potential mechanisms underlying the association between OSAHS and arteriosclerosis are complex. It is still unclear whether OSAHS directly affects arteriosclerosis or exerts its influence indirectly by causing related metabolic disturbances (46). We conducted a mediation analysis to clarify whether AHI affects arteriosclerosis through intermediate variables. The results showed that in T2DM patients, blood pressure indicators mediated the effect of AHI on arteriosclerosis. Although hypertension history and duration also had a mediating effect on the relationship, their impact was not significant. The direct effect of AHI on arteriosclerosis was also insignificant, which may indicate that AHI indirectly influences the increase in arterial stiffness by affecting blood pressure indicators rather than acting directly. OSAHS is closely related to hypertension, with the prevalence of hypertension in OSAHS patients being around 50-60% (47). Hypoxemia and hypercapnia in OSAHS trigger excessive activation of the sympathetic nervous system, and increased sympathetic activity leads to a sharp rise in catecholamine release, increasing cardiac output and peripheral vascular resistance, thereby causing nighttime blood pressure elevation (48). Notably, catecholamine levels in both serum and urine of OSAHS patients rise at night but persist even after the night, during waking periods. This prolonged elevation of catecholamines promotes the development of hypertension in OSAHS patients and is positively correlated with the severity of OSAHS (49, 50). In T2DM patients, hyperglycemia leads to the formation of a large number of advanced glycation end products, triggering oxidative stress, causing vasoconstriction, and contributing to the development of hypertension (51). Hypertension can further damage endothelial cells, increasing insulin resistance, accelerating lipid deposition on the vessel walls, and, through the impact of high-velocity blood flow on the vascular wall, leading to vascular injury and promoting the formation and progression of arteriosclerosis (52). Some experts point out that blood pressure variability, especially changes in SBP, is closely related to arteriosclerosis, and higher variability is associated with increased cardiovascular risk, while average blood pressure levels seem to have a less significant role in this regard (53). Studies have found that when OSAHS coexists with hypertension, there is a cumulative effect on arteriosclerosis, and the association between OSAHS and arteriosclerosis may be mediated by hypertension (54). Siwasaranond et al. (17) expressed a similar view, finding that the risk of arteriosclerosis in T2DM patients with severe OSAHS is 3.05 times higher than in those with mild or no OSAHS, and this relationship is mainly mediated by the presence of hypertension. Clearly, blood pressure control is a critical factor. We recommend personalized blood pressure management plans for T2DM patients with OSAHS through lifestyle interventions, medication adherence, and continuous monitoring with adjustments to the treatment plan as needed. In our study, although all patients were on relatively stable antihypertensive treatment, we did not meticulously document medication use, which may have somewhat obscured the role of blood pressure as a mediating variable. In future research, we can further validate this issue by incorporating ambulatory blood pressure monitoring.

This study has several limitations. First, it is a single-center retrospective study, and there may be selection bias in patient enrollment. Future research should consider expanding the sample scope and verifying it through prospective, multicenter studies to reflect the diversity of the target population more comprehensively. Second, the interactions between OSAHS, T2DM, and arteriosclerosis are complex and multifaceted, so we cannot completely rule out potential residual confounding factors, such as patients’ renal function, lifestyle, and medication use. In future studies, we recommend further incorporating relevant indicators to refine the analysis. Lastly, the study population was limited to T2DM patients, and these results may not broadly represent other populations.

## 6 Conclusions

OSAHS significantly impacts arteriosclerosis in T2DM patients, with blood pressure playing a partial mediating role in this process. Early identification and intervention of OSAHS, particularly at the mild OSAHS stage, may help reduce the risk of arteriosclerosis and associated cardiovascular events in T2DM patients, improving overall patient outcomes.

## Data Availability

The original contributions presented in the study are included in the article/supplementary material. Further inquiries can be directed to the corresponding author.
